# A nomogram for predicting malignant cerebral artery infarction in the modern thrombectomy era

**DOI:** 10.3389/fneur.2022.934051

**Published:** 2022-09-20

**Authors:** Wenting Guo, Jiali Xu, Wenbo Zhao, Mengke Zhang, Jin Ma, Jian Chen, Jiangang Duan, Qingfeng Ma, Haiqing Song, Sijie Li, Xunming Ji

**Affiliations:** ^1^Department of Neurology, Xuanwu Hospital, Capital Medical University, Beijing, China; ^2^Beijing Key Laboratory of Hypoxic Conditioning Translational Medicine, Xuanwu Hospital, Capital Medical University, Beijing, China; ^3^Department of Neurosurgery, Xuanwu Hospital, Capital Medical University, Beijing, China; ^4^Department of Emergency, Xuanwu Hospital, Capital Medical University, Beijing, China; ^5^Beijing Institute of Brain Disorders, Laboratory of Brain Disorders, Ministry of Science and Technology, Collaborative Innovation Center for Brain Disorders, Capital Medical University, Beijing, China

**Keywords:** ischemic stroke, thrombectomy, malignant, brain edema, nomogram

## Abstract

**Objective:**

This study aimed to develop and validate a nomogram to predict malignant cerebral artery infarction (MMI) after endovascular treatment (EVT) in patients with acute ischemic stroke (AIS) in the modern thrombectomy era.

**Methods:**

We retrospectively analyzed data from a prospective cohort of consecutive patients with AIS who underwent EVT at Xuanwu hospital between January 2013 and June 2021. A multivariable logistic regression model was employed to construct the nomogram for predicting MMI after EVT. The discrimination and calibration of the nomogram were assessed both in the derivation and validation cohorts.

**Results:**

A total of 605 patients were enrolled in this study, with 425 in the derivation cohort and 180 in the validation cohort. The nomogram was developed based on admission systolic blood pressure (SBP), the National Institute of Health Stroke Score (NIHSS), the Alberta Stroke Program Early Computed Tomography Score (ASPECTS), vessel occlusion site, EVT time window, and recanalization status. The nomogram displayed good discrimination with the area under the receiver operating characteristics (ROCs) curve (AUC) of 0.783 [95% confidence interval (*CI*), 0.726–0.840] in the derivation cohort and 0.806 (95% *CI*, 0.738–0.874) in the validation cohort. The calibration of the nomogram was good as well, with the Hosmer–Lemeshow test of *p* = 0.857 in the derivation cohort and *p* = 0.275 in the validation cohort.

**Conclusion:**

In the modern thrombectomy era, a nomogram containing admission SBP, NIHSS, ASPECTS, vessel occlusion site, EVT time window, and recanalization status may predict the risk of MMI after EVT in patients with AIS.

## Introduction

Malignant middle cerebral artery infarction (MMI) accounts for 30–50% of strokes caused by large vessel occlusion with a mortality of nearly 80% (1–3). Early decompressive hemicraniectomy (DHC) is the only effective treatment that can reduce mortality and improve clinical outcomes for patients with MMI (4). Thus, the rapid recognition of patients at high risk of MMI is helpful for timely clinical decisions.

Endovascular therapy (EVT) has shown overwhelming efficacy for AIS secondary to large vessel occlusion in the anterior circulation. This includes both early EVT (within 6 h) and late EVT (within 6–24 h) (5–7). However, MMI is common in patients undergoing EVT with an incidence of 19.2–44%, which lessens the benefits of EVT (8–14). Therefore, it is crucial to identify risk factors mediating the development of MMI after EVT and construct a predictive model specifically for this population.

Several studies have explored predictors of MMI in patients with AIS who underwent EVT in the early time window, two of which constructed a nomogram to predict MMI after early EVT (8–12, 14). However, these studies scarcely include patients selected by rigorous imaging examination who received EVT in the late time window. Therefore, this study intends to construct and validate an applicable nomogram predictive model to rapidly and accurately recognize patients at high risk of MMI after EVT in the modern thrombectomy era.

## Methods

### Study design and population

We retrospectively analyzed data from a prospective cohort of consecutive patients with AIS who underwent EVT at Xuanwu Hospital between January 2013 and June 2021. This prospective cohort was approved by the Ethics Committee of Capital Medical University Xuanwu Hospital (No. [2017]030), and written informed consent was obtained from all patients or their legal representatives.

The following criteria were required for participation in this study: (1) age ≥18 years; (2) AIS caused by occlusion of the internal carotid artery (ICA) or M1 and M2 segments of the middle cerebral artery (MCA); (3) EVT performed within 24 h from symptom onset; (4) confirmation of the diagnosis of MMI or non-MMI within 5 days after EVT; and (5) the absence of parenchymal hemorrhage type 2. All EVT procedures and management of patients followed the recommendation of the current guideline (15).

### Data collection

Demographics data (age and sex), vascular risk factors (hypertension, diabetes, hyperlipidemia, atrial fibrillation, and previous stroke), baseline characteristics [admission systolic blood pressure (SBP) and diastolic blood pressure (DBP)], admission stroke severity [the National Institute of Health Stroke Scale (NIHSS) and the Alberta Stroke Program Early Computed Tomography Score (ASPECTS)], vessel occlusion site (ICA occlusion or MCA occlusion), admission laboratory tests [low-density lipoprotein (LDL), fast blood glucose (FBG), and white blood cells (WBC)], stroke etiology [according to the Trial of Org 10,172 in Acute Stroke Treatment (TOAST) classification], treatment information [anesthesia, time interval from symptoms onset to puncture (OTP), time interval from symptoms onset to recanalization (OTR), passes of retriever, and recanalization status], and clinical outcomes at 90 days were collected from the database. Endovascular treatment initiated within 6 h from stroke symptom OTP was defined as the early time window (early EVT) and that within 6–24 h as the late time window (late EVT). Successful recanalization was defined as a modified Thrombolysis in Cerebral Infarction (mTICI) scale of 2b or 3. Clinical outcomes were assessed using the modified Rankin Scale (mRS) at 90 days through the telephone by trained staff, and a favorable outcome was defined as an mRS of 0–2.

### Definition of MMI

Malignant middle cerebral artery infarction or non-MMI was diagnosed within 5 days after the EVT procedure in combination with clinical and imaging characteristics. Patients with the following characteristics can be diagnosed as MMI (4): (1) NIHSS > 15 with a decreased level of consciousness (Item 1a ≥1) either on admission or after secondary deterioration, (2) infarction size exceeding at least two-thirds of the MCA territory on one of the follow-up head computed tomography or magnetic resonance imaging (MRI, within 5 days after EVT) with compression of the ventricles or midline shift, and (3) no other cause of neurological deterioration. The diagnosis of MMI was reviewed by two trained neurologists independently, and disagreement was resolved by discussion or consultation with a third neurologist.

### Statistics

Statistical analyses were performed using SPSS (Version 26; IBM Corp, Armonk, NY) and R Project for Statistical Computing (Version 4.1.2). The *p-*values of <0.05 (two-sided) were considered significant. Patients were randomly divided at a ratio of 6:4 into the derivation cohort and the validation cohort. Differences between the derivation cohort and the validation cohort were analyzed. Categorical variables are presented as numbers (percentages) and continuous variables are expressed as mean ± standard deviation (SD) or median [interquartile range (IQR)]. Differences between the two groups were explored using the chi-square test for categorical variables and the independent *t*-test or the Mann–Whitney *U*-test for continuous variables. Patients with missing data for the baseline characteristics were excluded from the study.

To construct a nomogram, we compared the differences between patients with and without MMI in the derivation cohort, then we implemented multivariable logistic regression analysis to determine independent predictors of MMI, including variables with a *p*-value of <0.1 in univariate analyses. Variance inflation factors (VIF) were calculated to assess the collinearity assumption (VIF <5 being considered non-significant). The odds ratio (*OR*) and 95% confidence interval (*CI*) of each significant risk factor in the final logistic regression model were calculated. Performances of the nomogram were assessed in the derivation and validation cohorts. Discrimination of the nomogram was assessed using the area under the receiver operating characteristics (ROCs) curve (AUC). An AUC of 0.51–0.7 indicates low accuracy, 0.71–0.9 indicates moderate accuracy, and 0.91–1.0 indicates high accuracy to discriminate between patients with MMI and without MMI. The calibration of the nomogram was evaluated by the Hosmer–Lemeshow goodness-of-fit test (*p* > 0.05 indicates good calibration).

## Results

**Figure 1:**
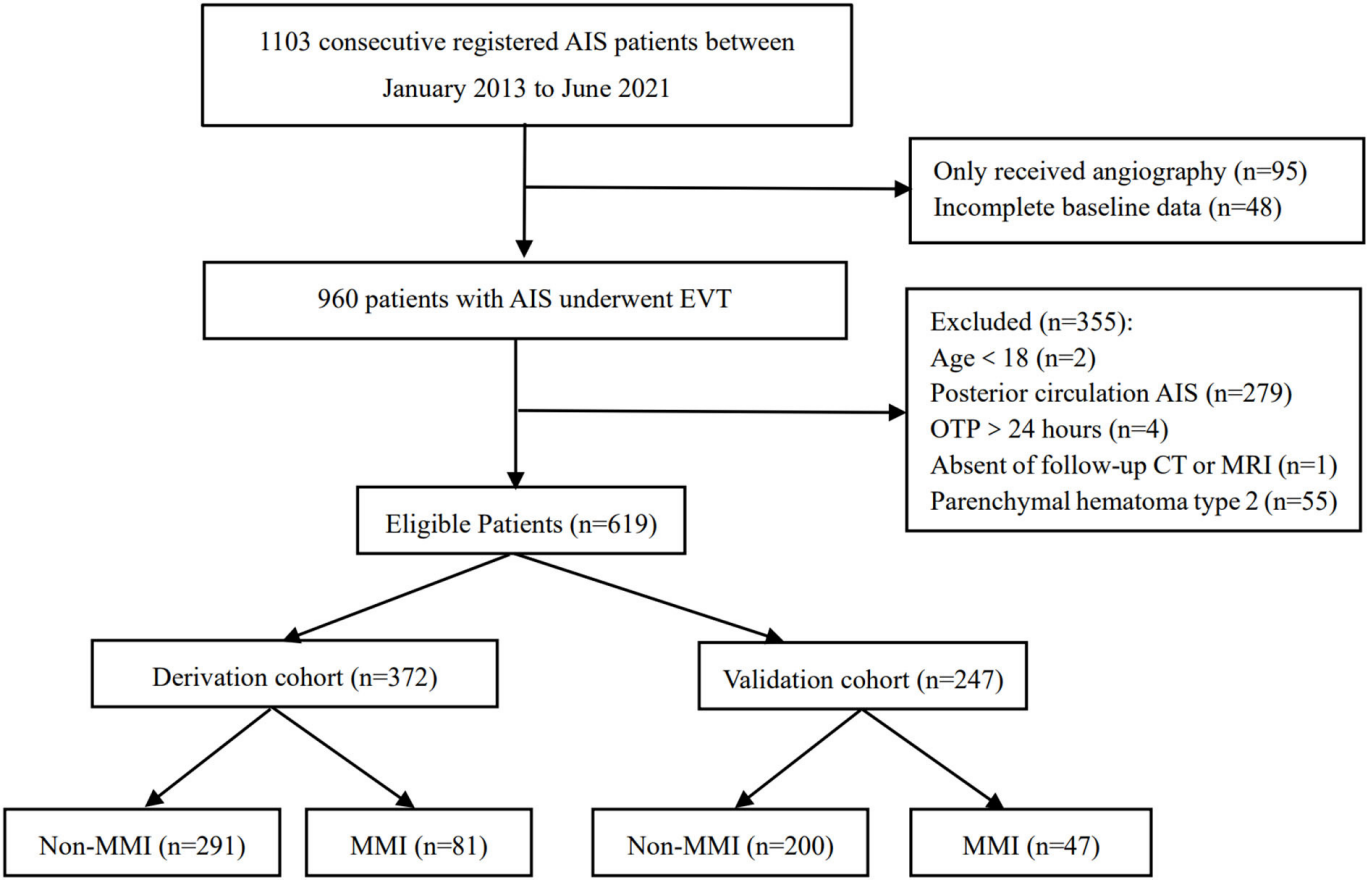
A flowchart of the patients enrolled in the study. AIS, acute ischemic stroke; EVT, endovascular therapy; OTP, time interval from symptoms onset to puncture; MMI, malignant middle cerebral artery infarction.

Between January 2013 and June 2021, this cohort registered a total of 1,103 patients. After excluding those patients who only received angiography (*n* = 95) and had incomplete baseline data (*n* = 48), 960 patients with AIS who underwent EVT were retrospectively screened (2 with age <18 years old, 279 with posterior circulation AIS, 4 with OTP >24 h, 1 absent of follow-up CT or MRI, and 55 with parenchymal hemorrhage type 2). Finally, 605 patients were included in the present study with 372 patients in the derivation cohort and 247 patients in the validation cohort (Figure 1). There was no significant difference in characteristics between the two cohorts except for the probability of successful recanalization (Table 1).

**Table 1:** Characteristics of patients in the derivation and validation cohorts.

**Factors**	**Derivation cohort**	**Validation cohort**	***P*-value**
	**(*n* = 372)**	**(*n* = 247)**	
**Demographics**			
Age (years), mean (SD)	64.5 ± 12.8	64.2 ± 13.3	0.771
Male, *n* (%)	245 (65.9%)	165 (66.8%)	0.808
**Vascular risk factors**, ***n*** **(%)**			
Hypertension	240 (64.5%)	168 (68.0%)	0.368
Diabetes	97 (26.1%)	65 (26.3%)	0.947
Hyperlipidemia	186 (50.0%)	131 (53.0%)	0.459
Atrial fibrillation	143 (38.4%)	98 (39.7%)	0.758
Previous stroke	91 (24.5%)	52 (21.1%)	0.324
**Baseline characteristics**			
Admission SBP (mmHg), mean ± SD	146.3 ± 24.5	144.6 ± 21.8	0.378
Admission DBP (mmHg), mean ± SD	83.6 ± 14.7	83.4 ± 13.5	0.866
**Stroke severity**			
Admission NIHSS, median (IQR)	15 (7)	15 (6)	0.990
Admission ASPECTS, median (IQR)	9 (2)	9 (3)	0.679
**Vessel occlusion site**			
ICA occlusion, *n* (%)	166 (44.6%)	103 (41.7%)	0.472
MCA occlusion, *n* (%)	206 (55.4%)	144 (58.3%)	
**Laboratory test**			
LDL (mmol/L), mean ± SD	2.7 ± 1.0	2.6 ± 0.9	0.162
FBG (mmol/L), median (IQR)	7.2 (3.2)	7.1 (2.9)	0.332
WBC (10^6^/L), mean ± SD	8.9 ± 2.8	8.6 ± 2.7	0.150
**Stroke etiology**			
LAA, *n* (%)	197 (53.0%)	128 (51.8%)	0.615
CE, *n* (%)	156 (41.9%)	110 (44.5%)	
Others, *n* (%)	19 (5.1%)	9 (3.6%)	
**Treatment**			
General anesthesia, *n* (%)	111 (29.8%)	77 (31.2%)	0.723
IVT, *n* (%)	145 (39.0%)	97 (39.3%)	0.942
OTP (min), median (IQR)	358 (243)	362 (211)	0.774
Early EVT, OTP <6 h, *n* (%)	187 (50.3%)	120 (48.6%)	0.681
Late EVT, OTP ≥6 h, *n* (%)	185 (49.7%)	127 (51.4%)	
OTR (min), median (IQR)	426 (226)	441 (212)	0.993
Passes of retriever ≥3, *n* (%)	49 (13.2%)	33 (13.4%)	0.946
Recanalization, *n* (%)	320 (86.0%)	228 (92.3%)	0.016
**MMI**, ***n*** **(%)**	81 (21.8%)	47 (10.0%)	0.409
**DHC**, ***n*** **(%)**	28 (7.5%)	11 (4.5%)	0.123
**Clinical outcome** ^$^			
mRS at 90 days, median (IQR)	3 (3)	3 (3)	0.716
Independence at 90 days, *n* (%)	160 (44.4%)	101 (42.8%)	0.692
Mortality at 90 days, *n* (%)	63 (17.5%)	40 (16.9%)	0.862

SBP, systolic blood pressure; DBP, diastolic blood pressure; NIHSS, National Institute of Health Stroke Scale; ASPECTS, Alberta Stroke Program Early Computed Tomography Score; ICA, internal carotid artery; MCA, middle cerebral artery; LDL, low-density lipoprotein; FBG, fast blood glucose; WBC, white blood cell; LAA, large artery atherosclerosis; CE, cardioembolism; OTP, time interval from symptoms onset to puncture; EVT, endovascular treatment; OTR, time interval from symptoms onset to recanalization; IVT, intravenous thrombolysis; MMI, malignant middle cerebral artery infarction; DHC, decompressive hemicraniectomy; mRS, modified Rankin scale.

*p <0.05.

$ Data of clinical outcomes at 90 days are available for 596 out of 605 patients, 360 patients were in the denervation cohort, and 236 were in the validation cohort.

### Univariate analyses of patients with or without MMI in the denervation cohort

In the derivation cohort, 81 patients developed MMI, and 291 did not. Univariate analyses of the derivation cohort (Table 2) showed that patients in the MMI group had higher admission SBP (152.9 ± 24.3 vs. 144.5 ± 24.3 mmHg, *p* = 0.006), had higher admission NIHSS (median 17 vs. 14, *p* < 0.001) and lower admission ASPECTS (median 9 vs. 9, *p* = 0.036), had higher WBC count [(9.5 ± 3.5) ×10^6^/L vs. (8.8 ± 2.7) ×10^6^/L, *p* = 0.060] and FBG (median, 7.9 vs. 7.1 mmol/L, *p* = 0.017) compared with the non-MMI group. Patients in the MMI group also had a higher proportion of ICA occlusion (67.9 vs. 38.1%, *p* < 0.001), had a higher proportion of general anesthesia (43.2 vs. 26.1%, *p* = 0.003), had passes of retriever ≥3 (23.5 vs. 10.3%, *p* = 0.002), and had a lower proportion of recanalization (70.4 vs. 90.4%, *p* < 0.001). Furthermore, the median OTP was 344 min in the MMI group and 364 min in the non-MMI group (*p* = 0.037), and patients with MMI were more likely to receive EVT in the early time window (58.0 vs. 48.1%, *p* = 0.114).

**Table 2:** Characteristics of patients with MMI and without MMI in the denervation cohort.

**Factors**	**MMI**	**Non-MMI**	***P*-value**
	**(*n* = 81)**	**(*n* = 291)**	
**Demographics**			
Age (years), mean ± SD	65.8 ± 13.8	64.2 ± 12.5	0.310
Male, *n* (%)	54 (66.7%)	191 (65.6%)	0.863
**Vascular risk factors**, *n* (%)			
Hypertension	56 (69.1%)	184 (63.2%)	0.326
Diabetes	23 (28.4%)	74 (25.4%)	0.591
Hyperlipidemia	41 (50.6%)	145 (49.8%)	0.900
Atrial fibrillation	33 (40.7%)	110 (37.8%)	0.630
Previous stroke	23 (28.4%)	68 (23.4%)	0.352
**Baseline characteristics**			
Admission SBP (mmHg), mean ± SD	152.9 ± 24.3	144.5 ± 24.3	0.006^*^
Admission DBP (mmHg), mean ± SD	85.9 ± 14.3	82.9 ± 14.8	0.113
**Stroke severity**			
Admission NIHSS, median (IQR)	17 (8)	14 (7)	<0.001^*^
Admission ASPECTS, median (IQR)	9 (3)	9 (2)	0.036^*^
**Vessel occlusion site**			
ICA occlusion, *n* (%)	55 (67.9%)	111 (38.1%)	<0.001^*^
MCA occlusion, *n* (%)	26 (32.1%)	180 (61.9%)	
**Laboratory test**			
LDL (mmol/L), mean ± SD	2.7 ± 0.9	2.7 ± 1.0	0.582
FBG (mmol/L), median (IQR)	7.9 (3.8)	7.1 (3.0)	0.017^*^
WBC (10^6^/L), mean ± SD	9.5 ± 3.5	8.8 ± 2.7	0.060
**Stroke etiology**			
LAA, *n* (%)	39 (48.1%)	158 (54.3%)	0.598
CE, *n* (%)	37 (45.7%)	119 (40.9%)	
Others, *n* (%)	5 (6.2%)	14 (4.8%)	
**Treatment**			
General anesthesia, *n* (%)	35 (43.2%)	76 (26.1%)	0.003^*^
IVT, *n* (%)	44 (54.3%)	183 (62.9%)	0.162
OTP (min), median (IQR)	344 (225)	364 (245)	0.045^*^
Early EVT, OTP <6 h, *n* (%)	47 (58.0%)	140 (48.1%)	0.114
Late EVT, OTP ≥6 h, *n* (%)	35 (42.2%)	183 (53.5%)	
OTR (min), median (IQR)	407 (209)	429 (222)	0.112
Passes of retriever ≥3, *n* (%)	19 (23.5%)	30 (10.3%)	0.002^*^
Recanalization, *n* (%)	57 (70.4%)	263 (90.4%)	<0.001^*^
**DHC**, ***n*** **(%)**	27 (33.3%)	1 (0.3%)	<0.001^*^
**Clinical outcome** ^$^			
mRS at 90 days, median (IQR)	5 (2)	2 (3)	<0.001^*^
Independence at 90 days, *n* (%)	4 (5.2%)	156 (55.1%)	<0.001^*^
Mortality at 90 days, *n* (%)	40 (51.9%)	23 (8.1%)	<0.001^*^

MMI, malignant middle cerebral artery infarction; SBP, systolic blood pressure; DBP, diastolic blood pressure; NIHSS, National Institute of Health Stroke Scale; ASPECTS, Alberta Stroke Program Early Computed Tomography Score; ICA, internal carotid artery; MCA, middle cerebral artery; LDL, low-density lipoprotein; FBG, fast blood glucose; WBC, white blood cell; LAA, large artery atherosclerosis; CE, cardioembolism; OTP, time interval from symptoms onset to puncture; EVT, endovascular treatment; OTR, time interval from symptoms onset to recanalization; IVT, intravenous thrombolysis; DHC, decompressive hemicraniectomy; mRS, modified Rankin scale.

* p <0.05.

$ Data of clinical outcomes at 90 days are available for 360 out of 372 patients, 283 were in the non-MMI group and 77 were in the MMI group.

### Predictors of MMI identified by multivariable logistic regression analysis

Predictors in the final multivariable logistic regression model (Table 3) included admission SBP (*OR*, 1.012; 95% *CI*, 1.001–1.023; *p* = 0.037), admission NIHSS (*OR*, 1.094; 95% *CI*, 1.047–1.143; *p* < 0.001), admission ASPECTS (*OR*, 0.720; 95% *CI*, 0.599–0.866; *p* < 0.001), ICA occlusion (*OR*, 2.996; 95% *CI*, 1.704–5.269; *p* < 0.001), late EVT (*OR*, 0.556; 95% *CI*, 0.308–1.001; *p* = 0.050), and recanalization (*OR*, 0.339; 95% *CI*, 0.170–0.673; *p* = 0.002).

**Table 3:** Multivariable logistic regression of possible predictors of MMI in the denervation cohort.

**Variable**	**β**	**SE**	**OR**	**95% CI**	***P*-value**
Admission NIHSS	0.090	0.022	1.094	1.047–1.143	<0.001
Admission ASPECTS	−0.328	0.094	0.720	0.599–0.866	<0.001
Admission SBP	0.012	0.006	1.012	1.001–1.023	0.037
ICA occlusion	1.097	0.288	2.996	1.704–5.269	<0.001
Late EVT	−0.588	0.300	0.556	0.308–1.001	0.050
Recanalization	−1.082	0.351	0.339	0.170–0.673	0.002

MMI, malignant middle cerebral artery infarction; NIHSS, National Institute of Health Stroke Scale; ASPECTS, Alberta Stroke Program Early Computed Tomography Score; SBP, systolic blood pressure; ICA, internal cerebral artery; EVT, endovascular treatment.

### Construction and validation of the nomogram

**Figure 2:**
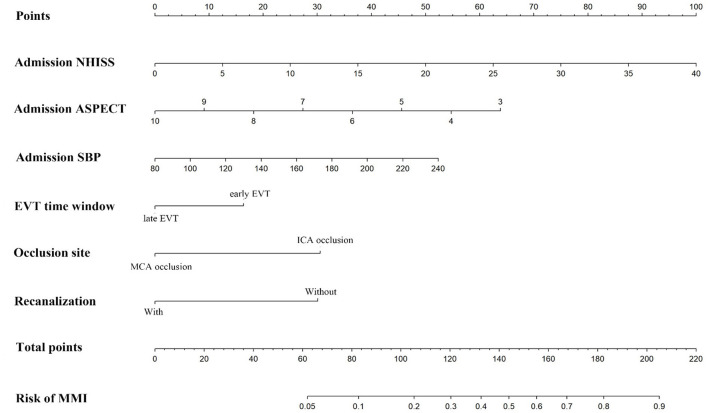
The nomogram for predicting the probability of malignant middle cerebral artery infarction. NIHSS, National Institute of Health Stroke Scale; ASPECTS, Alberta Stroke Program Early Computed Tomography Score; SBP, systolic blood pressure; EVT, endovascular therapy; ICA, internal carotid artery; MCA, middle cerebral artery; MMI, malignant middle cerebral artery infarction.

**Figure 3:**
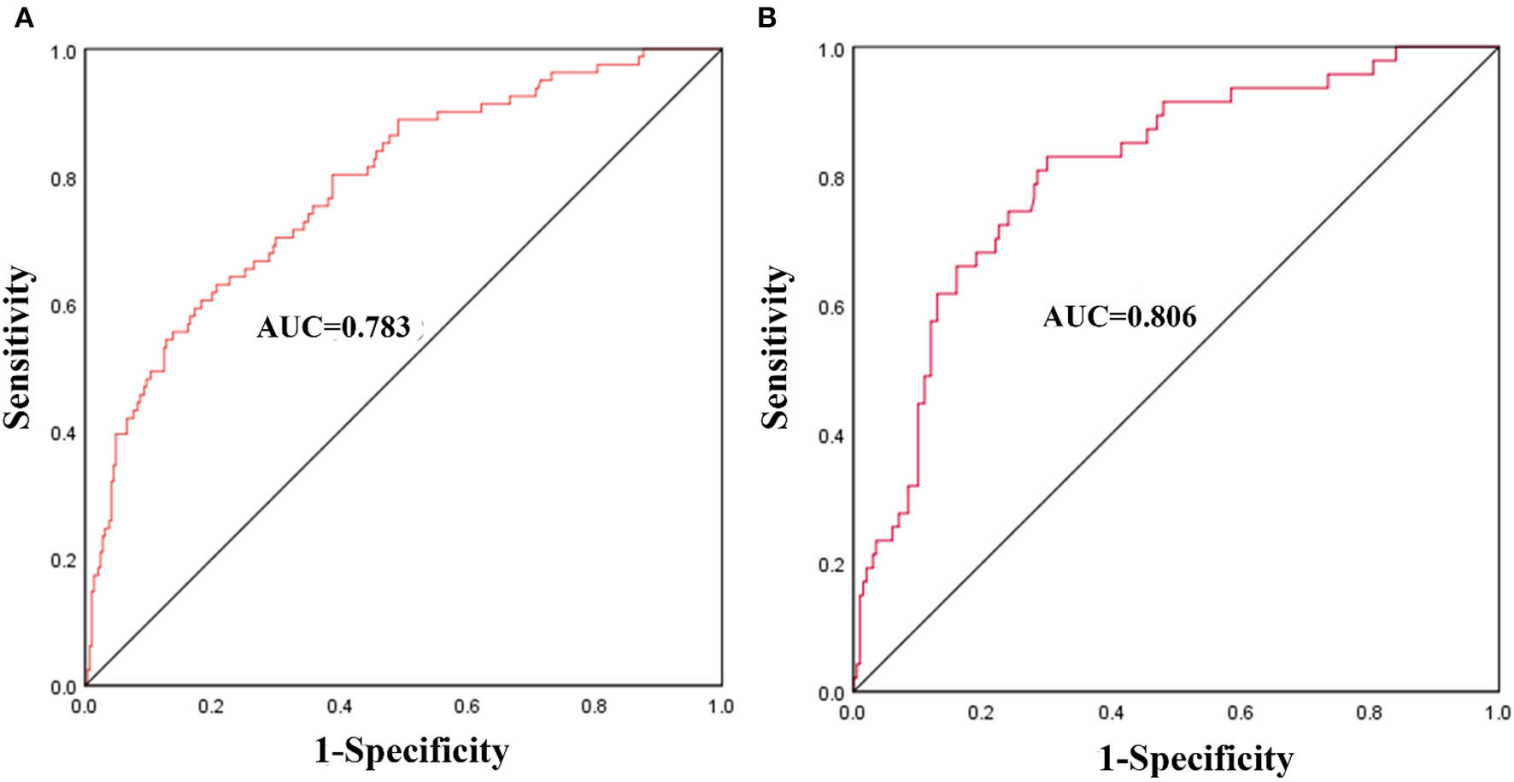
The receiver operating characteristics (ROCs) curve of the nomogram model to predict malignant middle cerebral artery infarction in the derivation cohort **(A)** and validation cohort **(B)**.

The nomogram was constructed based on admission SBP, NIHSS, ASPECTS, vessel occlusion site, EVT time window, and recanalization status (Figure 2). The discriminative ability of the nomogram was good with an AUC of 0.783 (95% *CI*, 0.726–0.840, Figure 3A) in the derivation cohort and 0.806 (95% *CI*, 0.738–0.874, Figure 3B) in the validation cohort. The calibration of this predictive nomogram was satisfactory both in the derivation cohort (the Hosmer–Lemeshow test, *p* = 0.857) and the validation cohort (the Hosmer–Lemeshow test, *p* = 0.257).

### MMI and neurological outcomes

In the derivation cohort, DHC was implemented in 27 (33.3%) patients in the MMI group and 1 (0.3%) patient in the non-MMI group. Data of clinical outcomes at 90 days were available for 360 out of 372 patients, 283 were in the non-MMI group and 77 were in the MMI group. Patients with MMI had a significantly higher proportion of mortality (51.9 vs. 8.1%, *p* < 0.001) and a lower proportion of favorable functional outcomes (5.2 vs. 55.1%, *p* < 0.001).

## Discussion

This study constructed and validated a practical nomogram based on admission SBP, NIHSS, ASPECTS, vessel occlusion site, EVT time window, and recanalization status, which displayed good discrimination and calibration to predict MMI after EVT in the modern thrombectomy era.

Predictive factors for MMI have been widely discussed. Consistent with previous studies, our study found that higher admission SBP, NIHSS, and lower ASPECTS predicted the occurrence of MMI (16, 17). In addition, we found that ICA occlusion was an independent predictor of MMI. Internal carotid artery occlusion implies fewer collaterals supplied from the anterior cerebral artery or anterior communication artery compared with MCA occlusion (10), and poor collaterals may augment the progression of infarct volume and lead to severe brain edema (18). Therefore, MMI occurred more frequently in patients with ICA occlusion. Successful recanalization has been demonstrated to decrease the incidence of MMI (10, 12, 17). Similarly, our study found that patients in the MMI group had a lower proportion of recanalization. Unexpectedly, we found that late EVT was associated with a lower risk of MMI. This can be explained by the rigorously selected patients for late EVT, namely, only those patients who had small or moderate infarct core and large salvageable brain tissue were eligible for late EVT. However, hyperglycemia and age, which were found to be strongly associated with MMI in some studies, were not independent predictors of MMI after EVT in our multivariable logistic regression model (8, 9).

Timely DHC can reduce mortality and improve functional outcomes for patients with MMI (4). However, patients with MMI had a lower proportion to receive DHC in real-world studies, because the decision on DHC was finally at the discretion of families of the patients (5, 13, 14). Furthermore, this study had a low percentage of DHC (33.3%) despite being higher than the percentage in previous similar studies (25.4, 16.7, and 9.7%) (5, 13, 14), which can be attributed to the observational design of the study. Thus, an accurate and simple predictive tool for MMI is crucial to facilitate the families of patients to understand the imperative of DHC for patients with MMI, thereby helping to enhance the proportion of DHC and to enable timely intervention. Previous studies have established several predictive models for MMI among patients with AIS, such as the EDEMA score (19), the DASH score (2), and the MBE score (20). However, these scoring systems were designed to distinguish potential MMI in patients with large hemisphere stroke, which were not specifically for patients who underwent EVT. Given that EVT could significantly decrease the incidence of malignant brain edema (10, 12, 17, 21), predictive models specifically designed for patients with EVT are imperative.

Du et al. established a nomogram that included age, baseline NIHSS, FBG, collateral circulation, and mTICI to predict malignant brain edema in patients undergoing EVT (9). Chen et al. developed a nomogram based on ASPECTS, hypertension, cisternal effacement, and recanalization status to predict the risk of malignant brain edema after EVT (14). However, these two studies only enrolled patients with AIS who underwent EVT within 6–8 h, and none of them has been validated externally. In the present study, we constructed and validated a nomogram consisting of admission SBP, NIHSS, ASPECTS, vessel occlusion site, EVT time window, and recanalization status. This nomogram had a moderate discriminative ability to predict MMI after EVT in the modern thrombectomy era as supported by the AUC value of 0.783 in the derivation cohort and 0.806 in the validation cohort. Besides, the calibration of this nomogram was good as well with *p* = 0.857 in the derivation cohort and *p* = 0.275 in the validation cohort. The performance of our nomogram model is not inferior to the nomogram of Du et al. and Chen et al., whose AUC was 0.805 and 0.874, respectively (9, 14). What is important, risk factors in our predictive model can be easily assessed as part of the routine clinical assessments for patients undergoing EVT.

Our study also has limitations. First, the results of our study were based on a single-center prospective registry, which might generate systematic bias and prevent the generalization of our predictive model. Second, some advanced predictors of MMI found in other studies, such as baseline infarct volume and collateral circulation, were not available in our study. Third, we excluded patients with parenchymal hemorrhage type 2, which might lead to selection biases due to the coexistence of malignant brain edema and hemorrhage in some patients. A multicenter study is necessary to confirm the performance of our nomogram model before its application in clinical practice. Finally, our study found that patients rigorously selected by imaging treated in the late time window had a lower risk of MMI, however, our cohort did not include data on perfusion mismatch volume that can be used for the exploration of the association between perfusion CT mismatch volume in the late therapeutic time window and the development of MMI.

## Conclusion

The nomogram composed of admission SBP, NIHSS, ASPECTS, vessel occlusion site, EVT time window, and recanalization status may predict the risk of MMI after EVT.

## Data availability statement

The raw data supporting the conclusions of this article will be available from the corresponding authors on reasonable request.

## Ethics statement

The studies involving human participants were reviewed and approved by Ethics Committee of Capital Medical University Xuanwu Hospital (No. [2017]030). The patients/participants provided their written informed consent to participate in this study.

## Author contributions

WG conceived of the study idea, collected and analyzed the data, and drafted the manuscript. JX, MZ, and JM participated in the data collection. WZ, SL, and XJ helped to modify the manuscript. XJ, JC, JD, QM, and HS participated in the coordination of the study. All authors read and approved the final manuscript.

## Funding

This study was supported by the Beijing Nova Program (No. Z201100006820143), the National Natural Science Foundation of China (Nos. 82001257, 81801313, and 81971114), and the General Project of Science and Technology of Beijing Municipal Education Commission (No. KM202110025018).

## Conflict of interest

The authors declare that the research was conducted in the absence of any commercial or financial relationships that could be construed as a potential conflict of interest.

## Publisher's note

All claims expressed in this article are solely those of the authors and do not necessarily represent those of their affiliated organizations, or those of the publisher, the editors and the reviewers. Any product that may be evaluated in this article, or claim that may be made by its manufacturer, is not guaranteed or endorsed by the publisher.
